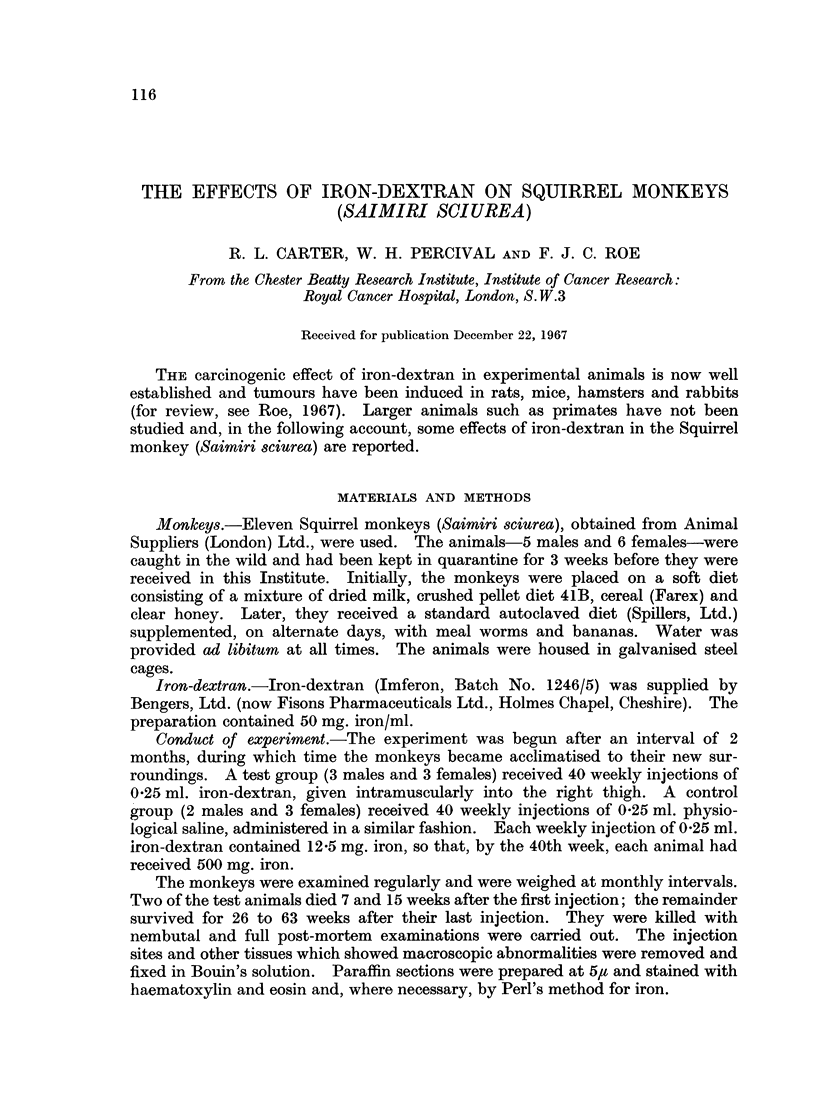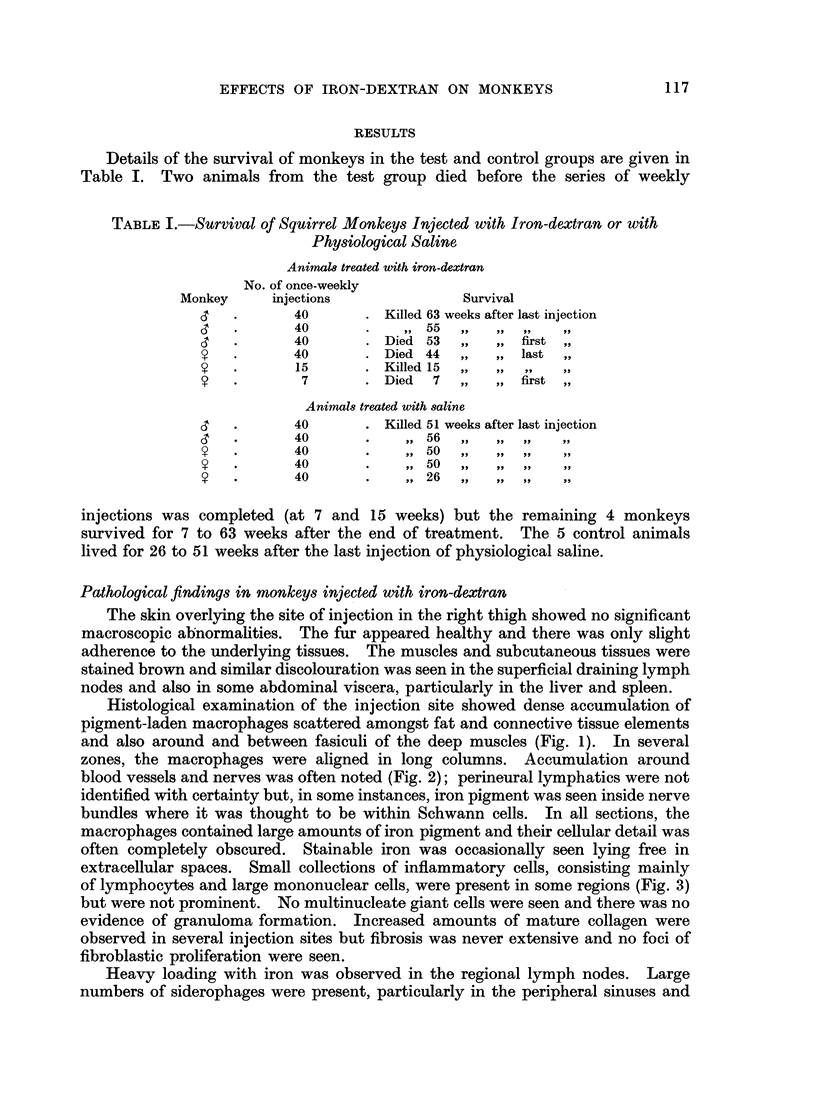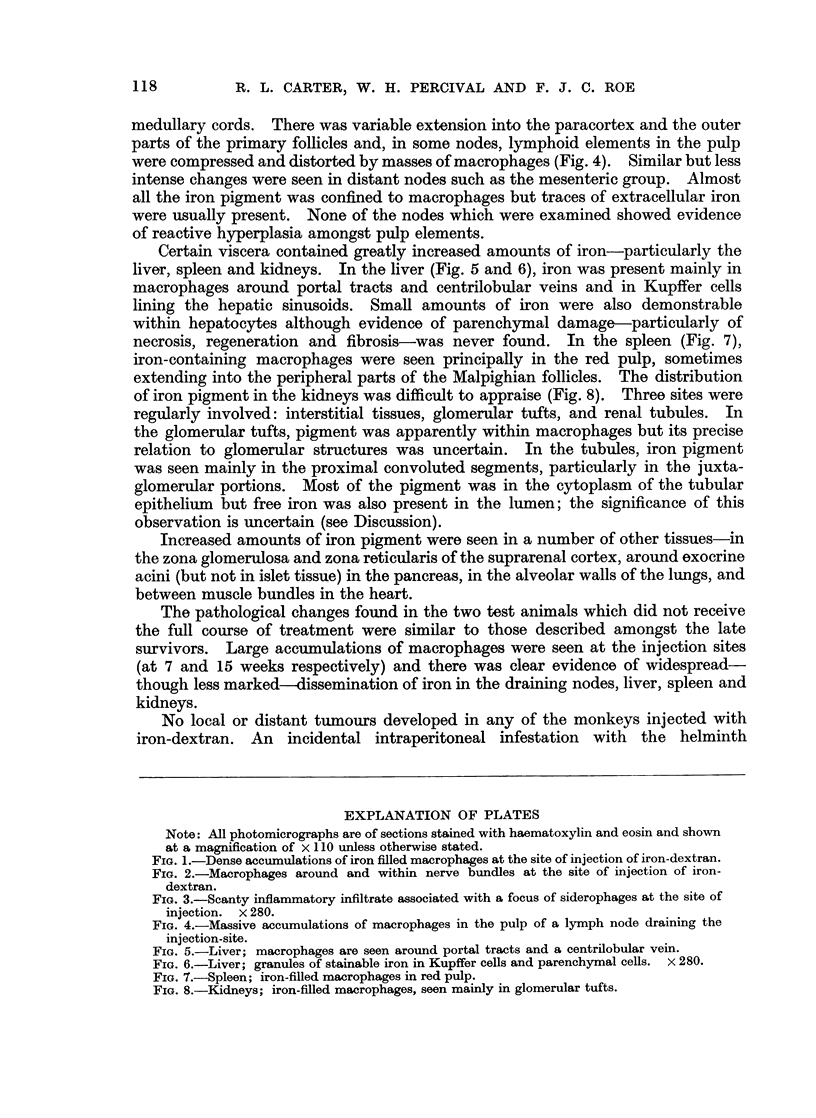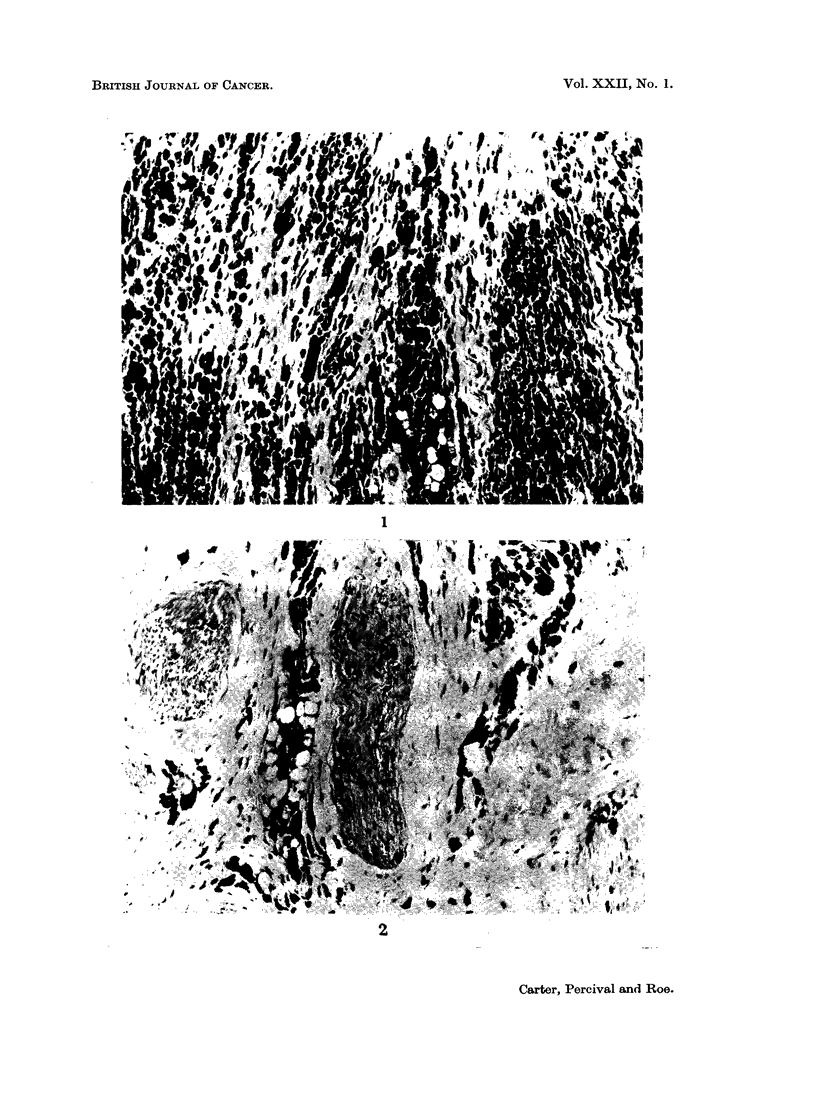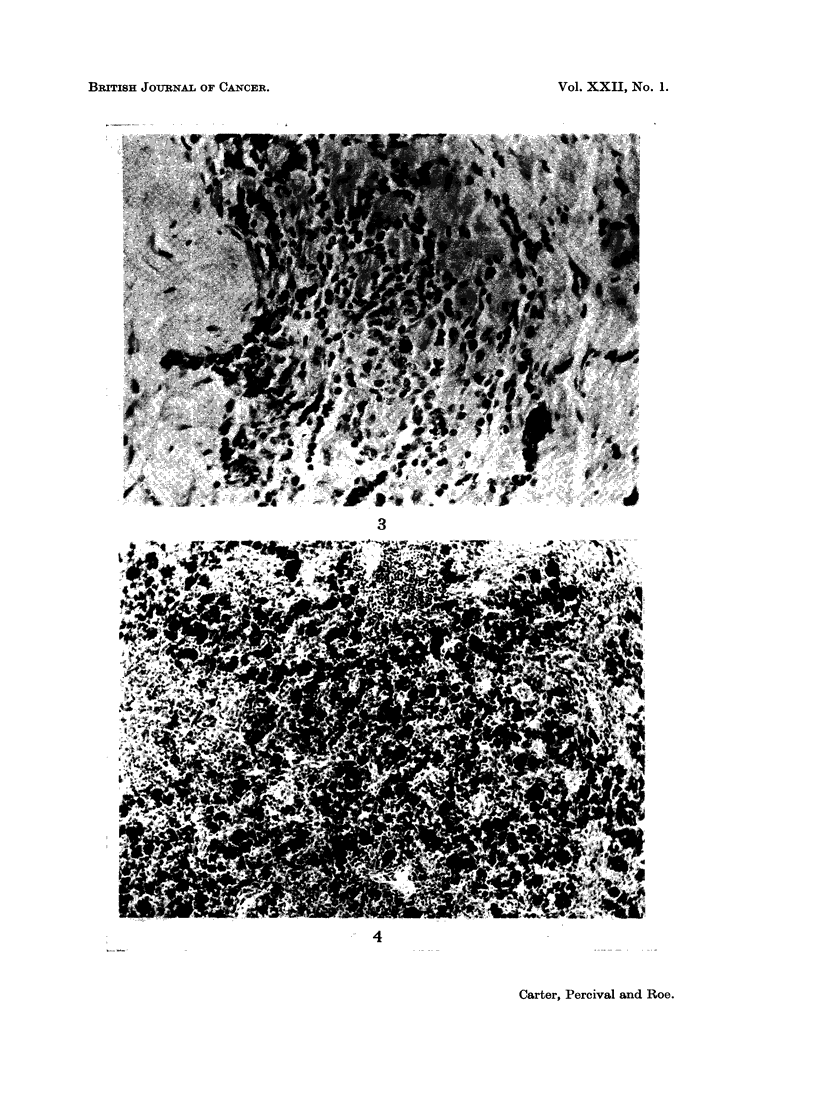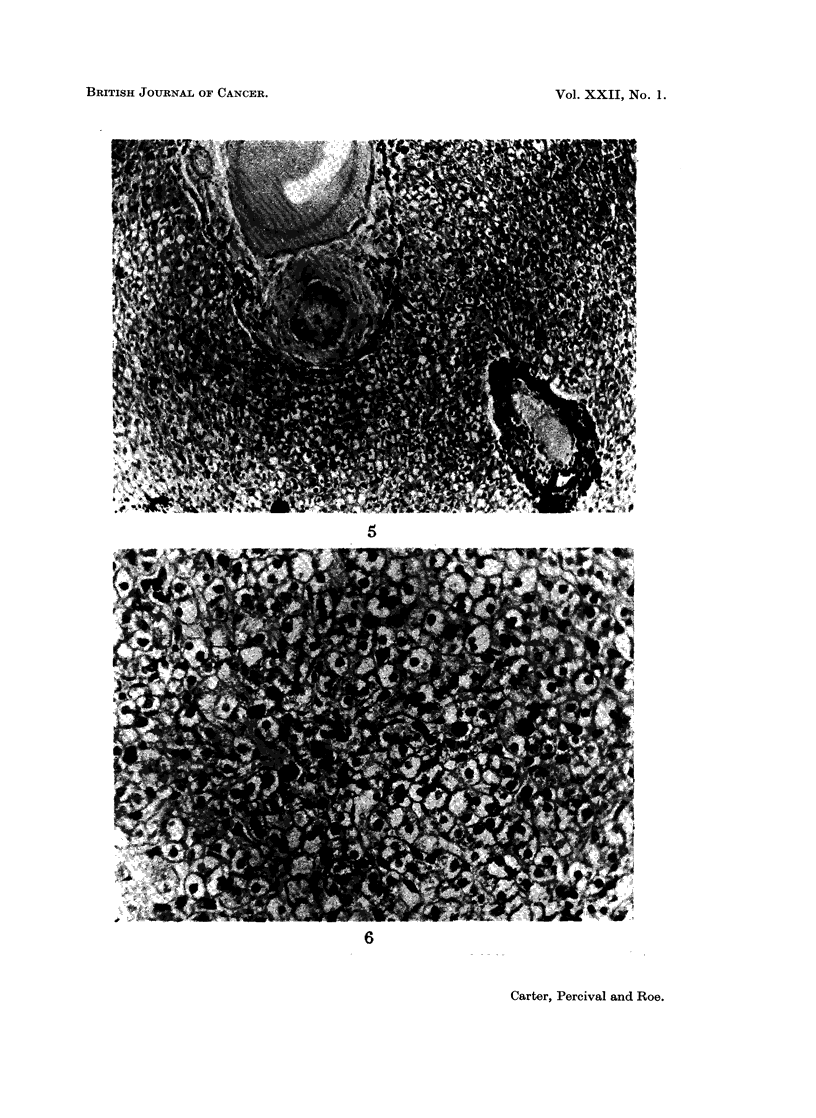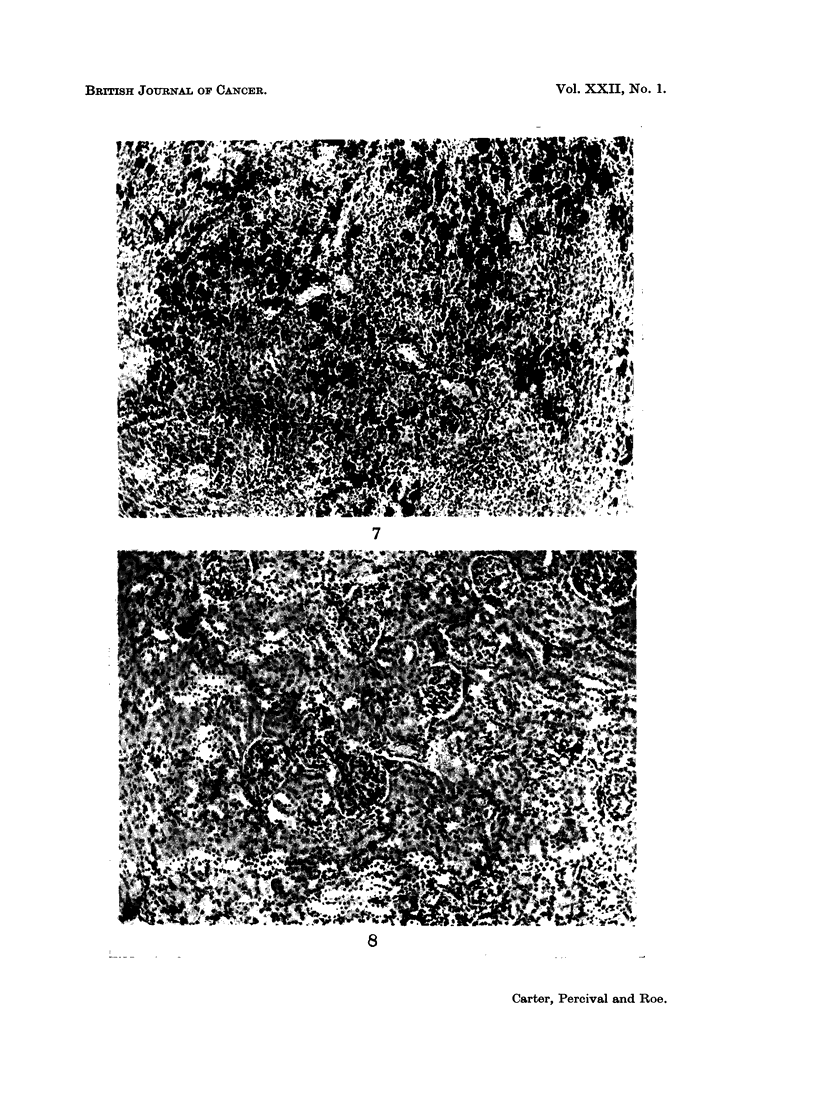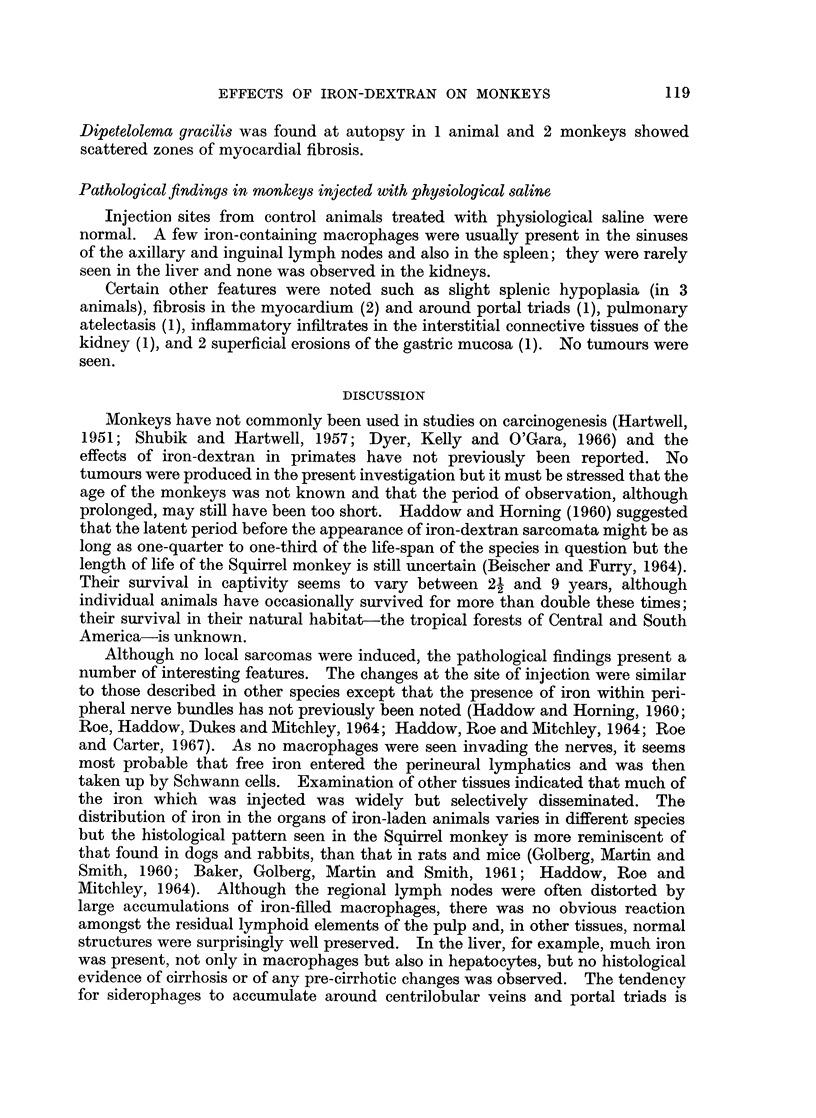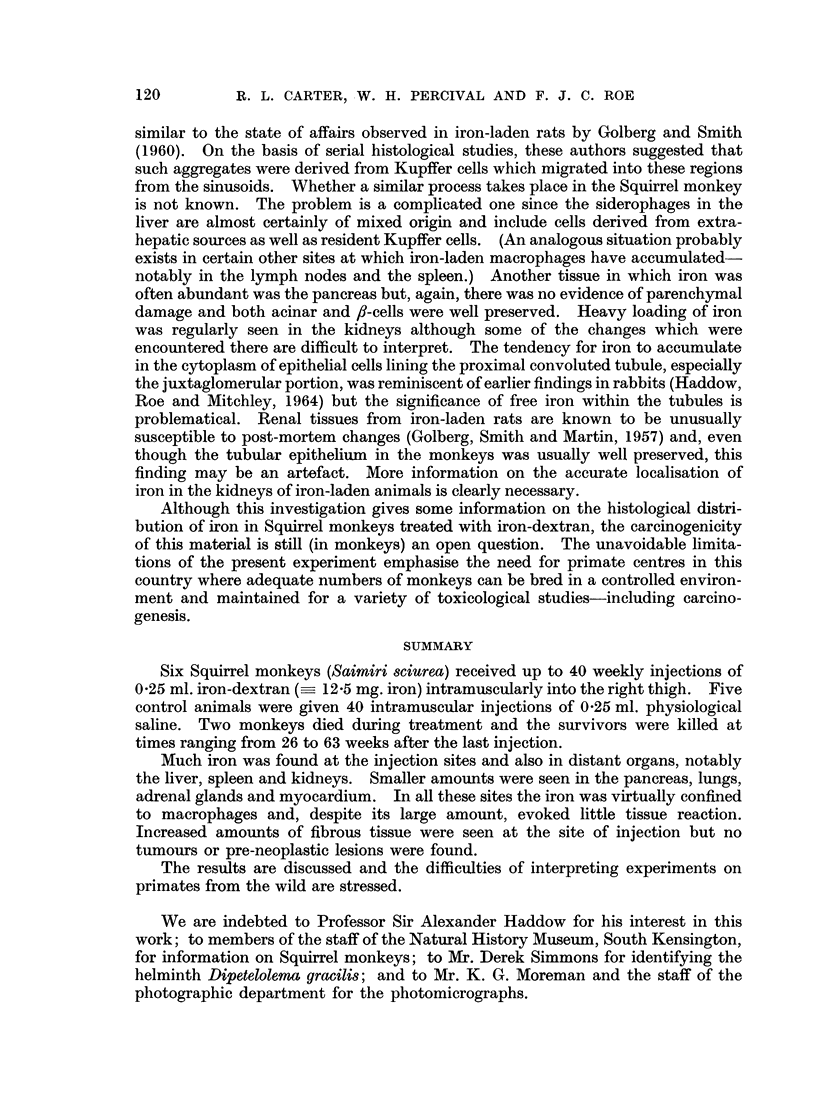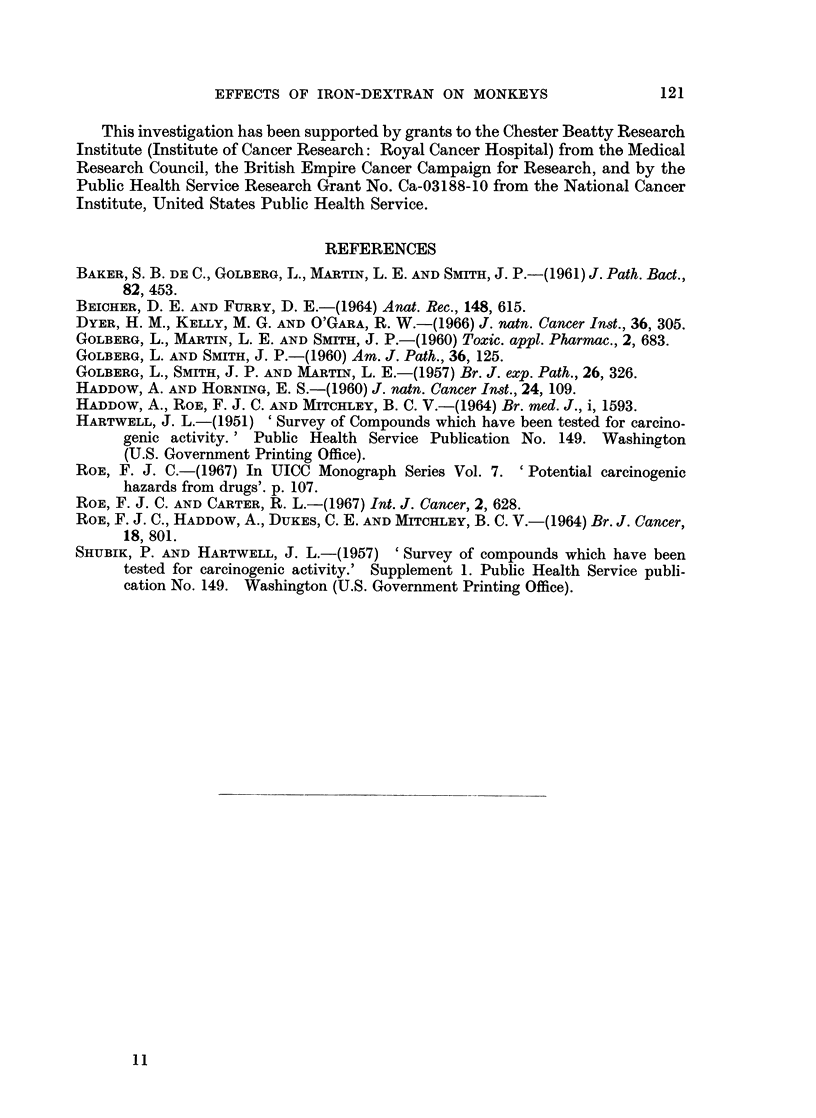# The effects of iron-dextran on squirrel monkeys (Saimiri sciurea).

**DOI:** 10.1038/bjc.1968.16

**Published:** 1968-03

**Authors:** R. L. Carter, W. H. Percival, F. J. Roe

## Abstract

**Images:**


### THE EFFECTS OF IRON-DEXTRAN ON SQUIRREL MONKEYS (*SAIMIRI SCIUREA*)

R. L. CARTER, W. H. PERCIVAL AND F. J. C. ROE

*From the Chester Beatty Research Institute, Institute of Cancer Research: Royal Cancer Hospital, London, S.W.3*

Received for publication December 22, 1967

THE carcinogenic effect of iron-dextran in experimental animals is now well established and tumours have been induced in rats, mice, hamsters and rabbits (for review, see Roe, 1967). Larger animals such as primates have not been studied and, in the following account, some effects of iron-dextran in the Squirrel monkey (*Saimiri sciurea*) are reported.

### MATERIALS AND METHODS

*Monkeys.*—Eleven Squirrel monkeys (*Saimiri sciurea*), obtained from Animal Suppliers (London) Ltd., were used. The animals—5 males and 6 females—were caught in the wild and had been kept in quarantine for 3 weeks before they were received in this Institute. Initially, the monkeys were placed on a soft diet consisting of a mixture of dried milk, crushed pellet diet 41B, cereal (Farex) and clear honey. Later, they received a standard autoclaved diet (Spillers, Ltd.) supplemented, on alternate days, with meal worms and bananas. Water was provided *ad libitum* at all times. The animals were housed in galvanised steel cages.

*Iron-dextran.*—Iron-dextran (Imferon, Batch No. 1246/5) was supplied by Bengers, Ltd. (now Fisons Pharmaceuticals Ltd., Holmes Chapel, Cheshire). The preparation contained 50 mg. iron/ml.

*Conduct of experiment.*—The experiment was begun after an interval of 2 months, during which time the monkeys became acclimatised to their new surroundings. A test group (3 males and 3 females) received 40 weekly injections of 0·25 ml. iron-dextran, given intramuscularly into the right thigh. A control group (2 males and 3 females) received 40 weekly injections of 0·25 ml. physiological saline, administered in a similar fashion. Each weekly injection of 0·25 ml. iron-dextran contained 12·5 mg. iron, so that, by the 40th week, each animal had received 500 mg. iron.

The monkeys were examined regularly and were weighed at monthly intervals. Two of the test animals died 7 and 15 weeks after the first injection; the remainder survived for 26 to 63 weeks after their last injection. They were killed with nembutal and full post-mortem examinations were carried out. The injection sites and other tissues which showed macroscopic abnormalities were removed and fixed in Bouin's solution. Paraffin sections were prepared at $5\mu$ and stained with haematoxylin and eosin and, where necessary, by Perl's method for iron.

RESULTS

Details of the survival of monkeys in the test and control groups are given in Table I. Two animals from the test group died before the series of weekly injections was completed (at 7 and 15 weeks) but the remaining 4 monkeys survived for 7 to 63 weeks after the end of treatment. The 5 control animals lived for 26 to 51 weeks after the last injection of physiological saline.

TABLE I.—*Survival of Squirrel Monkeys Injected with Iron-dextran or with Physiological Saline*

*Animals treated with iron-dextran*

| Monkey | No. of once-weekly injections | Survival |
|---|---|---|
| ♂ | 40 | Killed 63 weeks after last injection |
| ♂ | 40 | ,, 55 ,, ,, ,, ,, |
| ♂ | 40 | Died 53 ,, ,, first ,, |
| ♀ | 40 | Died 44 ,, ,, last ,, |
| ♀ | 15 | Killed 15 ,, ,, ,, ,, |
| ♀ | 7 | Died 7 ,, ,, first ,, |

*Animals treated with saline*

| Monkey | No. of once-weekly injections | Survival |
|---|---|---|
| ♂ | 40 | Killed 51 weeks after last injection |
| ♂ | 40 | ,, 56 ,, ,, ,, ,, |
| ♀ | 40 | ,, 50 ,, ,, ,, ,, |
| ♀ | 40 | ,, 50 ,, ,, ,, ,, |
| ♀ | 40 | ,, 26 ,, ,, ,, ,, |

*Pathological findings in monkeys injected with iron-dextran*

The skin overlying the site of injection in the right thigh showed no significant macroscopic abnormalities. The fur appeared healthy and there was only slight adherence to the underlying tissues. The muscles and subcutaneous tissues were stained brown and similar discolouration was seen in the superficial draining lymph nodes and also in some abdominal viscera, particularly in the liver and spleen.

Histological examination of the injection site showed dense accumulation of pigment-laden macrophages scattered amongst fat and connective tissue elements and also around and between fasiculi of the deep muscles (Fig. 1). In several zones, the macrophages were aligned in long columns. Accumulation around blood vessels and nerves was often noted (Fig. 2); perineural lymphatics were not identified with certainty but, in some instances, iron pigment was seen inside nerve bundles where it was thought to be within Schwann cells. In all sections, the macrophages contained large amounts of iron pigment and their cellular detail was often completely obscured. Stainable iron was occasionally seen lying free in extracellular spaces. Small collections of inflammatory cells, consisting mainly of lymphocytes and large mononuclear cells, were present in some regions (Fig. 3) but were not prominent. No multinucleate giant cells were seen and there was no evidence of granuloma formation. Increased amounts of mature collagen were observed in several injection sites but fibrosis was never extensive and no foci of fibroblastic proliferation were seen.

Heavy loading with iron was observed in the regional lymph nodes. Large numbers of siderophages were present, particularly in the peripheral sinuses and medullary cords. There was variable extension into the paracortex and the outer parts of the primary follicles and, in some nodes, lymphoid elements in the pulp were compressed and distorted by masses of macrophages (Fig. 4). Similar but less intense changes were seen in distant nodes such as the mesenteric group. Almost all the iron pigment was confined to macrophages but traces of extracellular iron were usually present. None of the nodes which were examined showed evidence of reactive hyperplasia amongst pulp elements.

Certain viscera contained greatly increased amounts of iron—particularly the liver, spleen and kidneys. In the liver (Fig. 5 and 6), iron was present mainly in macrophages around portal tracts and centrilobular veins and in Kupffer cells lining the hepatic sinusoids. Small amounts of iron were also demonstrable within hepatocytes although evidence of parenchymal damage—particularly of necrosis, regeneration and fibrosis—was never found. In the spleen (Fig. 7), iron-containing macrophages were seen principally in the red pulp, sometimes extending into the peripheral parts of the Malpighian follicles. The distribution of iron pigment in the kidneys was difficult to appraise (Fig. 8). Three sites were regularly involved: interstitial tissues, glomerular tufts, and renal tubules. In the glomerular tufts, pigment was apparently within macrophages but its precise relation to glomerular structures was uncertain. In the tubules, iron pigment was seen mainly in the proximal convoluted segments, particularly in the juxta-glomerular portions. Most of the pigment was in the cytoplasm of the tubular epithelium but free iron was also present in the lumen; the significance of this observation is uncertain (see Discussion).

Increased amounts of iron pigment were seen in a number of other tissues—in the zona glomerulosa and zona reticularis of the suprarenal cortex, around exocrine acini (but not in islet tissue) in the pancreas, in the alveolar walls of the lungs, and between muscle bundles in the heart.

The pathological changes found in the two test animals which did not receive the full course of treatment were similar to those described amongst the late survivors. Large accumulations of macrophages were seen at the injection sites (at 7 and 15 weeks respectively) and there was clear evidence of widespread—though less marked—dissemination of iron in the draining nodes, liver, spleen and kidneys.

No local or distant tumours developed in any of the monkeys injected with iron-dextran. An incidental intraperitoneal infestation with the helminth

---

EXPLANATION OF PLATES

Note: All photomicrographs are of sections stained with haematoxylin and eosin and shown at a magnification of $\times 110$ unless otherwise stated.

FIG. 1.—Dense accumulations of iron filled macrophages at the site of injection of iron-dextran.

FIG. 2.—Macrophages around and within nerve bundles at the site of injection of iron-dextran.

FIG. 3.—Scanty inflammatory infiltrate associated with a focus of siderophages at the site of injection. $\times 280$.

FIG. 4.—Massive accumulations of macrophages in the pulp of a lymph node draining the injection-site.

FIG. 5.—Liver; macrophages are seen around portal tracts and a centrilobular vein.

FIG. 6.—Liver; granules of stainable iron in Kupffer cells and parenchymal cells. $\times 280$.

FIG. 7.—Spleen; iron-filled macrophages in red pulp.

FIG. 8.—Kidneys; iron-filled macrophages, seen mainly in glomerular tufts.

1

2

3

4

Carter, Percival and Roe.

5

6

Carter, Percival and Roe.

7

8

*Dipetelolema gracilis* was found at autopsy in 1 animal and 2 monkeys showed scattered zones of myocardial fibrosis.

*Pathological findings in monkeys injected with physiological saline*

Injection sites from control animals treated with physiological saline were normal. A few iron-containing macrophages were usually present in the sinuses of the axillary and inguinal lymph nodes and also in the spleen; they were rarely seen in the liver and none was observed in the kidneys.

Certain other features were noted such as slight splenic hypoplasia (in 3 animals), fibrosis in the myocardium (2) and around portal triads (1), pulmonary atelectasis (1), inflammatory infiltrates in the interstitial connective tissues of the kidney (1), and 2 superficial erosions of the gastric mucosa (1). No tumours were seen.

### DISCUSSION

Monkeys have not commonly been used in studies on carcinogenesis (Hartwell, 1951; Shubik and Hartwell, 1957; Dyer, Kelly and O'Gara, 1966) and the effects of iron-dextran in primates have not previously been reported. No tumours were produced in the present investigation but it must be stressed that the age of the monkeys was not known and that the period of observation, although prolonged, may still have been too short. Haddow and Horning (1960) suggested that the latent period before the appearance of iron-dextran sarcomata might be as long as one-quarter to one-third of the life-span of the species in question but the length of life of the Squirrel monkey is still uncertain (Beischer and Furry, 1964). Their survival in captivity seems to vary between $2\frac{1}{2}$ and 9 years, although individual animals have occasionally survived for more than double these times; their survival in their natural habitat—the tropical forests of Central and South America—is unknown.

Although no local sarcomas were induced, the pathological findings present a number of interesting features. The changes at the site of injection were similar to those described in other species except that the presence of iron within peripheral nerve bundles has not previously been noted (Haddow and Horning, 1960; Roe, Haddow, Dukes and Mitchley, 1964; Haddow, Roe and Mitchley, 1964; Roe and Carter, 1967). As no macrophages were seen invading the nerves, it seems most probable that free iron entered the perineural lymphatics and was then taken up by Schwann cells. Examination of other tissues indicated that much of the iron which was injected was widely but selectively disseminated. The distribution of iron in the organs of iron-laden animals varies in different species but the histological pattern seen in the Squirrel monkey is more reminiscent of that found in dogs and rabbits, than that in rats and mice (Golberg, Martin and Smith, 1960; Baker, Golberg, Martin and Smith, 1961; Haddow, Roe and Mitchley, 1964). Although the regional lymph nodes were often distorted by large accumulations of iron-filled macrophages, there was no obvious reaction amongst the residual lymphoid elements of the pulp and, in other tissues, normal structures were surprisingly well preserved. In the liver, for example, much iron was present, not only in macrophages but also in hepatocytes, but no histological evidence of cirrhosis or of any pre-cirrhotic changes was observed. The tendency for siderophages to accumulate around centrilobular veins and portal triads is similar to the state of affairs observed in iron-laden rats by Golberg and Smith (1960). On the basis of serial histological studies, these authors suggested that such aggregates were derived from Kupffer cells which migrated into these regions from the sinusoids. Whether a similar process takes place in the Squirrel monkey is not known. The problem is a complicated one since the siderophages in the liver are almost certainly of mixed origin and include cells derived from extra-hepatic sources as well as resident Kupffer cells. (An analogous situation probably exists in certain other sites at which iron-laden macrophages have accumulated—notably in the lymph nodes and the spleen.) Another tissue in which iron was often abundant was the pancreas but, again, there was no evidence of parenchymal damage and both acinar and $\beta$-cells were well preserved. Heavy loading of iron was regularly seen in the kidneys although some of the changes which were encountered there are difficult to interpret. The tendency for iron to accumulate in the cytoplasm of epithelial cells lining the proximal convoluted tubule, especially the juxtaglomerular portion, was reminiscent of earlier findings in rabbits (Haddow, Roe and Mitchley, 1964) but the significance of free iron within the tubules is problematical. Renal tissues from iron-laden rats are known to be unusually susceptible to post-mortem changes (Golberg, Smith and Martin, 1957) and, even though the tubular epithelium in the monkeys was usually well preserved, this finding may be an artefact. More information on the accurate localisation of iron in the kidneys of iron-laden animals is clearly necessary.

Although this investigation gives some information on the histological distribution of iron in Squirrel monkeys treated with iron-dextran, the carcinogenicity of this material is still (in monkeys) an open question. The unavoidable limitations of the present experiment emphasise the need for primate centres in this country where adequate numbers of monkeys can be bred in a controlled environment and maintained for a variety of toxicological studies—including carcinogenesis.

### SUMMARY

Six Squirrel monkeys (*Saimiri sciurea*) received up to 40 weekly injections of 0·25 ml. iron-dextran ($\equiv$ 12·5 mg. iron) intramuscularly into the right thigh. Five control animals were given 40 intramuscular injections of 0·25 ml. physiological saline. Two monkeys died during treatment and the survivors were killed at times ranging from 26 to 63 weeks after the last injection.

Much iron was found at the injection sites and also in distant organs, notably the liver, spleen and kidneys. Smaller amounts were seen in the pancreas, lungs, adrenal glands and myocardium. In all these sites the iron was virtually confined to macrophages and, despite its large amount, evoked little tissue reaction. Increased amounts of fibrous tissue were seen at the site of injection but no tumours or pre-neoplastic lesions were found.

The results are discussed and the difficulties of interpreting experiments on primates from the wild are stressed.

We are indebted to Professor Sir Alexander Haddow for his interest in this work; to members of the staff of the Natural History Museum, South Kensington, for information on Squirrel monkeys; to Mr. Derek Simmons for identifying the helminth *Dipetelolema gracilis*; and to Mr. K. G. Moreman and the staff of the photographic department for the photomicrographs.

This investigation has been supported by grants to the Chester Beatty Research Institute (Institute of Cancer Research: Royal Cancer Hospital) from the Medical Research Council, the British Empire Cancer Campaign for Research, and by the Public Health Service Research Grant No. Ca-03188-10 from the National Cancer Institute, United States Public Health Service.

### REFERENCES

BAKER, S. B. DE C., GOLBERG, L., MARTIN, L. E. AND SMITH, J. P.—(1961) *J. Path. Bact.*, **82**, 453.

BEICHER, D. E. AND FURRY, D. E.—(1964) *Anat. Rec.*, **148**, 615.

DYER, H. M., KELLY, M. G. AND O'GARA, R. W.—(1966) *J. natn. Cancer Inst.*, **36**, 305.

GOLBERG, L., MARTIN, L. E. AND SMITH, J. P.—(1960) *Toxic. appl. Pharmac.*, **2**, 683.

GOLBERG, L. AND SMITH, J. P.—(1960) *Am. J. Path.*, **36**, 125.

GOLBERG, L., SMITH, J. P. AND MARTIN, L. E.—(1957) *Br. J. exp. Path.*, **26**, 326.

HADDOW, A. AND HORNING, E. S.—(1960) *J. natn. Cancer Inst.*, **24**, 109.

HADDOW, A., ROE, F. J. C. AND MITCHLEY, B. C. V.—(1964) *Br. med. J.*, i, 1593.

HARTWELL, J. L.—(1951) 'Survey of Compounds which have been tested for carcinogenic activity.' Public Health Service Publication No. 149. Washington (U.S. Government Printing Office).

ROE, F. J. C.—(1967) In UICC Monograph Series Vol. 7. 'Potential carcinogenic hazards from drugs'. p. 107.

ROE, F. J. C. AND CARTER, R. L.—(1967) *Int. J. Cancer*, **2**, 628.

ROE, F. J. C., HADDOW, A., DUKES, C. E. AND MITCHLEY, B. C. V.—(1964) *Br. J. Cancer*, **18**, 801.

SHUBIK, P. AND HARTWELL, J. L.—(1957) 'Survey of compounds which have been tested for carcinogenic activity.' Supplement 1. Public Health Service publication No. 149. Washington (U.S. Government Printing Office).